# Recent progress in perovskite solar cells: the perovskite layer

**DOI:** 10.3762/bjnano.11.5

**Published:** 2020-01-06

**Authors:** Xianfeng Dai, Ke Xu, Fanan Wei

**Affiliations:** 1 School of Information & Control Engineering, Shenyang Jianzhu University, Shenyang, China; 2 School of Mechanical Engineering and Automation, Fuzhou University, Fuzhou, China

**Keywords:** coating techniques, perovskite layer, perovskite solar cells (PSCs), perovskite structure, photovoltaic technology, scalability

## Abstract

Perovskite solar cells (PSCs) are set to be game changing components in next-generation photovoltaic technology due to their high efficiency and low cost. In this article, recent progress in the development of perovskite layers, which are the basis of PSCs, is reviewed. Achievements in the fabrication of high-quality perovskite films by various methods and techniques are introduced. The reported works demonstrate that the power conversion efficiency of the perovskite layers depends largely on their morphology and the crystalline quality. Furthermore, recent achievements concerning the scalability of perovskite films are presented. These developments aim at manufacturing large-scale perovskite solar modules at high speed. Moreover, it is shown that the development of low-dimensional perovskites plays an important role in improving the long-term ambient stability of PSCs. Finally, these latest advancements can enhance the competitiveness of PSCs in photovoltaics, paving the way for their commercialization. In the closing section of this review, some future critical challenges are outlined, and the prospect of commercialization of PSCs is presented.

## Review

### Introduction

**Figure 1:**
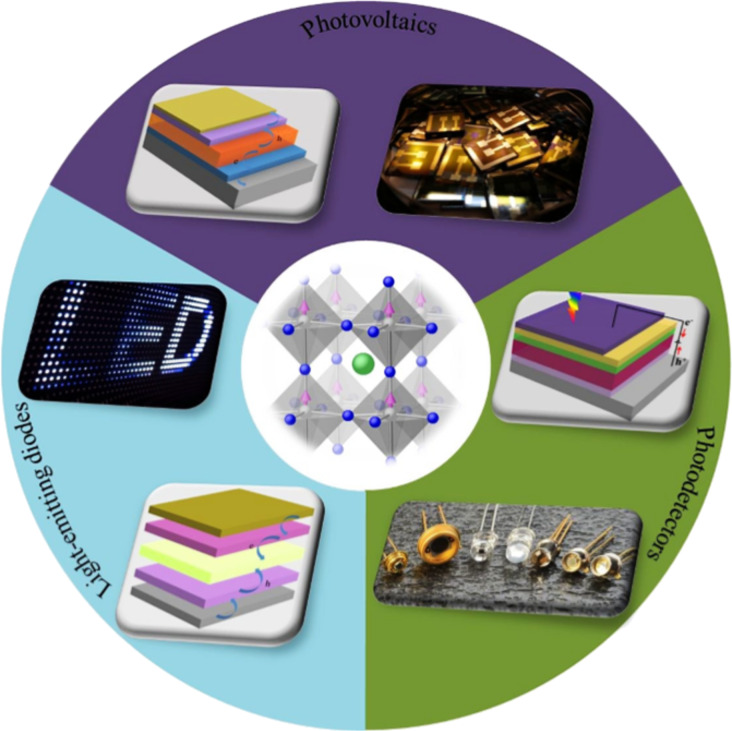
Schematic diagram showing an OIHP crystal (center) and its applications in various optoelectronic devices such as photovoltaics, light-emitting diodes and photodetectors. Reproduced with permission from [63], copyright 2019 Royal Society of Chemistry.

During the past decade, organic–inorganic halide perovskites (OIHP) have attracted great interest due to their special merits, including exciting optical properties, outstanding optical tunability and low-temperature solution processability. Recently, OIHPs have been developed into solar cells, photodetectors and light-emitting diodes (Figure 1). In OIHP photovoltaics, perovskite solar cells (PSCs) have entered our field of vision. With their high efficiency and low cost, they are expected to be highly influential in next-generation photovoltaic technology. In recent years, with the introduction of a variety of strategies and the application of new techniques [1–6], the power conversion efficiency (PCE) records of PSCs are constantly being beat. While writing this review article, the highest certified PCE reached 25.2% [7], which is comparable to that of recent commercial photovoltaic modules. However, the poor long-term ambient stability and the weak scalability of PSCs have been critical hurdles for their commercialization. In general, the competitiveness of solar cells is evaluated by efficiency, cost and lifetime, where scalability is closely related to cost and lifetime has much to do with stability. Achieving a balance of efficiency, cost and lifetime is the key to promote the commercialization of PSCs to grab a share of the energy market. Therefore, several research efforts are addressing these issues that are important for commercialization. A few unprecedented achievements have been made that are highly beneficial for the large-scale commercial application of PSCs in the future [8–11].

For PSCs, the perovskite layer, which is sandwiched between an electron extraction layer and a hole extraction layer, is fundamental. It is the core of PSCs, which is closely related to the performance of the overall device. For example, it has been widely recognized that the PCE of the PSCs depends largely on the morphology and the crystalline quality of the perovskite layer [12]. Therefore, recent major challenges are to simultaneously control the film morphology and the crystalline quality and to prepare high-quality perovskite films with complete surface coverage, uniform morphology and large well-crystallized grains [13–16]. At the same time, research also focuses on the preparation of large-area perovskite films of high quality to obtain large-area perovskite solar modules [17–18]. It has also become a new research focus to improve the stability of PSCs by adjusting the structure of the perovskite [19–20].

In this article, the progress of PSC development is reviewed, concentrating on the perovskite functional layer, and valuable insights are provided. Moreover, the main challenges in the commercialization of PSCs are pointed out and possible solutions are discussed. Finally, the major challenges to be faced in the future are outlined and some brief outlook is presented.

### Fabrication of high-quality perovskite films

Benefiting from the advances of various film deposition techniques, such as one-step spin coating [13,21], two-step sequential solution deposition [22–23] and inkjet printing fabrication [24–25], unprecedented progress in the improvement of the efficiency of PSCs has been made. To obtain high-quality perovskite films based on these techniques, researchers control the perovskite morphology by optimizing the solvents for processing [26], varying the annealing temperature [27], adjusting the processing additives [28], sophisticated engineering of the solvent [12,14] and annealing of the solvent [29–30]. Here, some common and effective methods are introduced.

#### Additives

As is now well known, introducing additives to perovskite precursor solutions is a common way to optimize the film morphology and enhance the crystalline quality. Wang et al. [31] demonstrated a method to achieve fast formation and crystallization of perovskite films by incorporating hydrobromic acid (HBr) into the perovskite precursor solutions. Here, the halogen ion, a strong donor, can interact strongly with Pb^2+^ to form a homogeneous solution, which is beneficial for the swift growth of high-quality films. A perovskite solar cell with lower leakage current, better surface coverage and a PCE of 15.76% was fabricated. The addition of HBr shortened the crystallization time. However, the grains of the perovskite films were still not sufficiently large and dense to reach higher PCEs.

In order to solve this problem, Bao et al. [28] introduced formamidine acetate salt (FAAc) as an additive to optimize both the crystalline quality and the film morphology. By this, highly homogeneous perovskite films with low trap states and uniform morphology could be fabricated. Also, it has been proved that the addition of FAAc can effectively improve the charge transport efficiency by eliminating the defect and trap density in the perovskite film. The resulting planar PSCs doped with 5 mol % FAAc achieved a PCE of 18.90%, which corresponds to an enhancement of the PCE of over 20% compared to those fabricated by doping with HBr. These results imply that additives can effectively optimize the film morphology and enhance the crystalline quality to improve the performance of PSCs. A variety of additives with better quality are still sought. Additive engineering is not just one of the simplest and cheapest ways to improve the PCE, moreover, it is a universal method applicable in various perovskite deposition processes.

#### Solvent engineering

Recently, solvent engineering is considered to be the most popular method applied in one-step deposition processes. Nazeeruddin et al. [32] independently developed an anitisolvent deposition approach based on one-step spin coating, which involves dripping a noncoordinate solvent such as toluene, chlorobenzene, dichlorobenzene or trifluorotoluene onto the perovskite film while spinning. This method has shown to produce extremely smooth and homogeneous perovskite films. PSCs with a PCE of 20.3% were fabricated by using trifluorotoluene as the antisolvent. However, the application of trifluorotoluene, toluene and chlorobenzene in mass production is hindered because of their toxicity and the high cost. A nontoxic and inexpensive antisolvent is highly desired, which can regulate the perovskite crystallization process to fabricate films of superior quality and controllable morphology.

**Figure 2:**
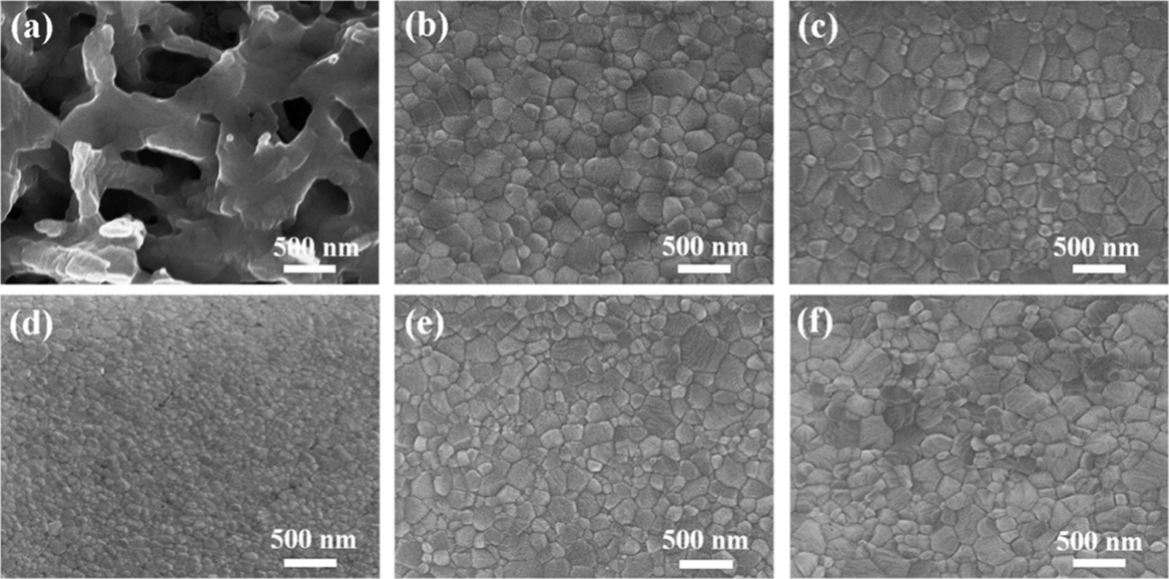
Scanning electron microscopy images of perovskite films prepared by the antisolvent deposition method treated with different solvents: (a) without antisolvent, (b) toluene, (c) chlorobenzene, (d) chloroform, (e) diethyl ether and (f) di-isopropyl ether. Reproduced with permission from [13], copyright 2017 Royal Society of Chemistry.

Deng et al. [13] produced efficient PSCs with diisopropyl ether as the antisolvent, which perfectly overcome this barrier. As shown in Figure 2, perovskite films fabricated by an antisolvent deposition process, where large perovskite crystals pack densely without pinholes, exhibit a smoother and more uniform morphology compared to those prepared by the traditional one-step method without antisolvent treatment. Perovskite films fabricated using diisopropyl ether treatment have larger crystallites compared to those fabricated using other solvents including toluene, chlorobenzene, chloroform and diethyl ether. The results demonstrate that solvent engineering, which is on track to break PCE records, is a more promising method compared to other methods of optimizing film morphology and crystalline quality. More importantly, the method is also simple and cost-efficient. Solvent engineering is expected to advance the development of economic, efficient large-scale PSCs .

#### New deposition techniques

Wong et al. [33] proposed a novel approach for the formation of perovskite films called “one and a half”-step (or 1.5-step) deposition, which combines the benefits of both one-step and sequential deposition. In the first step, an initial perovskite phase is obtained by using a mesh-assisted grid deposition technique. Subsequently, the deposited perovskite grid is dipped in formamidinium iodide-isopropanol to convert it into the proper perovskite phase, which is termed the “half step”. Efficient semi-transparent PSCs with a PCE of 10.03% and an average visible transmittance (AVT) of 28% were achieved. The devices prepared by the 1.5-step process show better performance than those prepared by the one-step process owing to their improved crystal quality, crystal size, absorbance and stability. Furthermore, the perovskite films fabricated by the mesh-assisted grid technique are of controllable transparency and have shown the potential to be scalable. These attributes provide a promising route for the development of large-scale semi-transparent PSCs with large area, having diverse applications in the field of building-integrated photovoltaics (BIPVs) or as top cells for tandem devices.

It has become a problem that a considerable amount of toxic Pb-containing material is discarded during the spin coating and spray coating PSC fabrication processes. Jiang et al. [34] solved this problem by successfully applying inkjet printing to deposit a flat and uniform CH_3_NH_3_PbI_3_ (MAPbI_3_) perovskite layer on a TiO_2_ film. Inkjet printing is a noncontact printing technique with direct control of material deposition, which can lead to an overall reduction of material usage and waste. Mathies et al. [24] report the fabrication and optimization of multipass inkjet-printed PSCs. Here, the thickness and grain size of the perovskite films were controlled during multipass inkjet printing of a MAPbI_3_-ink yielding PSCs with a high PCE of up to 11.3%. Although the PCE of inkjet-printed PSCs is lower than that of PSCs fabricated by spin coating or spray coating, inkjet printing is an environmentally friendly technique. With these preliminary results, we demonstrate a major step towards highly efficient, low-cost PSCs producing less Pb waste, thus promoting the development of perovskite digital printing technologies.

#### Large-area fabrication of perovskite films

**Figure 3:**
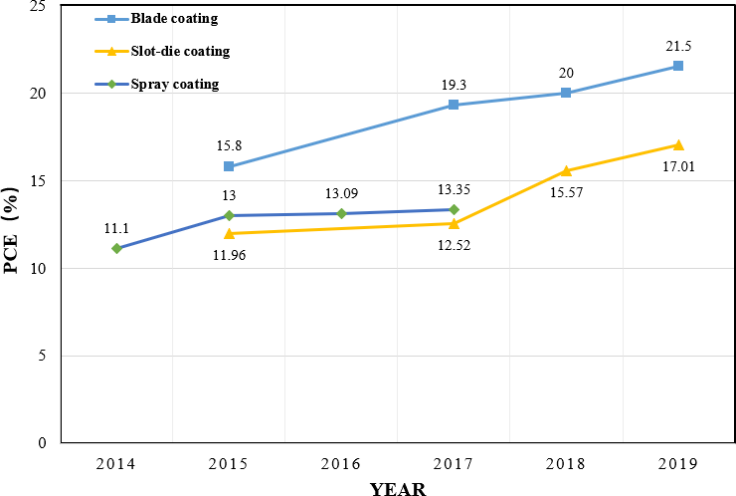
Progress of the efficiency of recently reported large-area PSCs fabricated by various deposition methods. The data is taken from [35–51].

Since the PCE of PSCs already exceeds 23%, the research focus is gradually shifting towards important issues related to commercialization. One of the challenges met is the fabrication of large-area perovskite solar modules. Establishing scalable deposition processes of perovskite materials is a prerequisite for the preparation of large-area modules. The quality and the uniformity of perovskite films based on spin coating decrease sharply with the increase of the area. Hence, a variety of scalable perovskite deposition processes, including blade coating, slot-die coating and spray coating, are proposed [35–42]. The photovoltaic parameters of recently reported large-area PSCs are listed in Table 1. The improvement of their efficiency over time is summarized in Figure 3.

**Table 1:** Summary of relevant photovoltaic parameters of recently reported large-area PSCs.

Aperture area [cm^2^]	Active area [cm^2^]	*V*_OC_ [V]	*J*_SC_ [mA cm^−2^]	FF [%]	PCE [%]	Device structure	Processing method	Ref.

16	–	1.13	17.3	61.9	12.1	FTO/bl-TiO_2_/m-TiO_2_/(HC(NH_2_)_2_PbI_3_)_0.85_ (CH_3_NH_3_PbBr_3_)_0.15_/spiro-OMeTAD/Au	spray coating	[43]
1.1	–	1.14	22.0	80	20.0	ITO/PTAA/BAA-modified perovskite/C_60_/bathocuproine (BCP)/Cu	blade coating	[46]
1.96	–	1.09	22.64	73.66	18.3	FTO/compact TiO_2_/mesoporous TiO_2_/MAPbI_3_/Co(II)- and Co(III)-based porphyrins/Au	blade coating	[17]
0.1	–	–	–	–	20.5			
33.0	–	–	–	–	15.3	ITO/PTAA/MAPbI_3_/fullerene (C_60_)/bathocuproine (BCP)/Cu	blade coating	[36]
57.2	–	–	–	–	14.6			
–	0.075	1.12	22.6	81.0	20.3			
–	roll to roll	1.10	17.21	67.25	12.7	ITO/ZnO NPs/MAPbI_3_/biﬂuo-OMeTAD/MoO_3_/Ag	slot-die coating	[48]
–	roll to roll	1.02	19.79	77.15	15.57	ITO/m-PEDOT:PSS/MAPbI_3_/PC_61_BM/Ca/Al	slot-die coating	[37]
–	roll to roll	0.99	17.39	64.82	11.16			
–	100	1.12	21.50	71.0	17.01	FTO/SnO_2_/MAPbI_3_/spiro-OMeTAD/Al	D-bar coating	[49]

#### Spray coating

Spray coating is the fastest method of obtaining scalable perovskite layers providing a high coating rate in a continuous process. For the first time, Lidzey et al. [43] used the ultrasonic spray coating technique to coat a precursor solution containing MAI and PbCl_2_ over the solar cell substrate. Here, the champion devices exhibit a PCE of 11.1%. This report demonstrates the possibility of spray coating for large-area deposition of perovskite films. Sanjib et al. [44] proved that controlling the solar cell substrate temperature could enhance the coverage of the resulting perovskite layers. The possibility of applying the spray-coating technique to the roll-to-roll fabrication process for highly efficient, flexible large-area PSCs has been demonstrated. It is widely believed that by the use of spray coating a two-step deposition of precursor solutions is well achievable. Yun et al. [45] presented a new spray-coating process based on antisolvent crystallization. Their so-called spray-antisolvent (SAS) technique facilitates the fabrication of large-area perovskite films of high quality. Here, PSCs with a stabilized PCE of 12.1% and large dimension of 16 cm^2^ are produced by combining the advantages of SAS and the use of a metal grid. The mentioned studies pave the way towards highly efficient large-area PSCs. However, spray coating is not frequently used since it is usually performed under ambient conditions, which may perturb the sensitive precursor materials.

#### Blade coating

Blade coating is a broadly adopted method of fabricating scalable perovskite films, which has shown the greatest potential among all the different coating methods, particularly for PSCs with an area larger than 10 cm^2^. Blade coating is a relatively simple method, where the perovskite precursor solution is usually swiped over a preheated substrate by a blade to obtain perovskite films after solvent evaporation. Many studies have demonstrated that the quality of the resulting perovskite films can be improved by controlling the substrate temperature [46–47]. In recent years, additives have also been used in blade coating to obtain compact perovskite films with fewer pinholes and uniform size distribution [17,48]. Huang et al. [36] formulated a perovskite ink that can dramatically improve the blade-coating quality of perovskite films at a high coating speed. Here, the dependence of the film thickness (*t*) on the speed of coating (*v*) was different for the two experimental modes tested. Namely, the blading speed in the so-called Landau–Levich mode is much faster than that in the so-called evaporation mode. Consequently, the Landau–Levich mode is more suitable for fast PSC production. However, at such high speeds, compact and uniform large-area perovskite films are not easily fabricated. This problem could be circumvented by incorporating very small amounts of surfactant additives such as ʟ-α-phosphatidylcholine into the perovskite ink solution. The surfactant additive improves the adhesion of the perovskite ink to hydrophobic substrates, effectively inhibiting the solution flow dynamics in the drying perovskite ink layer leading to compact and uniform perovskite films. The very small amount of surfactant additive has no adverse effect on the optoelectronic properties of the bladed films and can even passivate charge traps to further improve the device performance. Finally, stabilized PCEs of 15.3% and 14.6% were reached for devices with active areas of 33.0 cm^2^ and 57.2 cm^2^ at a high coating speed of 180 m·h^−1^. More importantly, the surfactants could be a kind of general additive to improve the quality of perovskite films and could be used in other scalable fabrication methods to enable the high-speed deposition of perovskite films. The strategy may open up new dimensions of the fabrication of large-area perovskite solar modules at a high speed and thus enhance the competitiveness of PCSs in photovoltaics.

#### Slot-die coating

Slot-die coating, which is essentially similar to the blade coating technique, is one of the most common roll-to-roll techniques. Ink made of a precursor solution is pumped into the coating head to form a continuous solution meniscus between the substrate and the coating head during the process. This is classified as a precoating technique, in which perovskite films with premetered thickness are prepared by controlling the processing parameters such as the coating speed, the length of the meniscus, the coating temperature and the pumping rate of the solution. However, it is not usually easy to obtain uniform, pinhole-free perovskite films, because the slow natural drying mechanism of the slot-die system, which allows the perovskite to be free in the wet film, causes the crystal to grow out of control. Ciro et al. [49] showed that the morphology and the thickness of the perovskite layers could be controlled by adjusting the coating temperature and the solution pumping rate. However, it is challenging to control the thickness and coverage only with these two parameters. To prevent the formation of overgrown crystals and pinholes, Kim et al. [50] introduced an external air-blowing system to the slot-die coating apparatus. The external air-blowing system is made up of a slot-die head releasing high-pressure nitrogen gas. Air-blowing simulates the effect of gas quenching encountered in the spin coating method. During the slot-die-coating step, gas quenching and substrate heating were combined to control the drying rate of the perovskite precursor solution, resulting in a uniform and compact high-quality perovskite layer. A PCE of 12.7% was achieved when a print friendly hole transport layer was introduced.

Recently, several remarkable milestones have been achieved concerning the preparation of the precursor solution. Ding et al. [37] incorporated an NH_4_Cl additive into the precursor solution accompanied by a mild substrate temperature and an air-blowing system to improve the crystallinity and the morphology of the perovskite films as well as the device performance. They produced solar cells with PCEs of 15.57%. In order to form uniform and high-quality perovskite films more quickly, Park et al. [51] invented a new coating solution for large-area perovskite films based on a MA-assisted MAPbI_3_ solution. The coating solutions contain preformed perovskite clusters, which can be rapidly (within 20 s) deposited on a substrate of an area larger than 100 cm^2^. The MAPbI_3_ films formed by the wire-bar (D-bar) coating technique were highly oriented. Smooth high-quality perovskite films with an average PCE of 17.01% were obtained because of the unique morphology, which resulted in long carrier lifetime and low defect density. Of course, this precursor solution can be used for slot-die coating to fabricate high-quality PCSs at a high throughput rate. The combination of this special coating solution and the slot-die-coating technology enables rapid and precise coating of films, and therefore is a promising method for large-scale PSC production at high yield.

### Stability of PSCs based on novel perovskite structures

The poor long-term stability of PSCs is another challenge for future commercial applications. Although PSCs have achieved a certificated PCE of 23.7%, the instability of traditional three-dimensional (3D) perovskites under ambient operational conditions, including weather, light, temperature and humidity changes, remains a critical hurdle for commercialization. The results of many efforts of several research groups to improve PSC stability [52–54] have been poor. In recent years, two-dimensional (2D) and 2D/3D perovskites have attracted great attention due to their superior moisture-resistance as well as the larger degrees of freedom of their chemistry and the much larger formation energy compared to 3D perovskites, which has shown to improve the stability of devices [55–56]. Table 2 summarizes the stability of some recently reported PSCs.

**Table 2:** Stability of selected recently reported PSCs.

Perovskite	Dimensionality	Device structure	PCE [%]	Stability	Ref.

(FAPbI_3_)_0.85_ (MAPbBr_3_)_0.15_	3D	FTO/mp-TiO_2_/perovskite/4,4-dimethoxytriphenylamine/Au	19.17	600 h stability at 40% RH	[51]
(FAPbI_3_)_0.95_ (MAPbBr_3_)_0.05_	3D	FTO/mp-TiO_2_/perovskite/DM/Au	21.7	95% left after 500 h	[52]
MA_2_PbI_4_	2DRP	FTO/TiO_2_/perovskite/spiro-OMeTAD/Au	16.6	97.8% left after 1512 h at 55% RH	[8]
(PDA)(MA)_3_Pb_4_I_13_	2DDJ	FTO/TiO_2_/perovskite/spiro-OMeTAD/Au	13.3	over 95% for 4000 h at 40–70% RH	[19]
(Cs_0.1_FA_0.9_)Pb(I_0.9_Br_0.1_)_3_	2D/3D	FTO/c-TiO_2_/m-TiO_2_/perovskite/spiro-OMeTAD/Au	20.08	92% left after 2400 h at 55% RH	[59]
Cs_0.05_(FA_0.83_MA_0.17_)_0.95_ Pb(I_0.83_Br_0.17_)_3_	2D/3D	FTO/SnO_2_/perovskite/spiro-OMeTAD/Au	21.06	90% left after 3000 h in air	[60]

#### 2D PSCs

The generic formula of 2D perovskites is (RNH_3_)_2_A*_n_*_-1_M*_n_*X_3_*_n_*_+1_ (*n* = 1, 2, 3, … ), where RNH_3_^+^ is a large aliphatic or aromatic alkylammonium cation, A^+^ is monovalent organic cation, M^2+^ is a divalent metal, and X^−^ is a halide anion [57]. The overall 2D structure is stabilized via van der Waals interactions. Importantly, the 2D perovskite structure can also be considered as a multiple-quantum-well structure, which obviously suppresses the ion migration that is evident in 3D perovskite. More importantly, these 2D perovskites offer environmental stability because of their unique properties like larger formation energy and hydrophobicity. Due to these special physical and chemical properties, 2D perovskites play a major role in improving the stability of PSCs under ambient conditions.

2D Ruddlesden–Popper (2DRP) perovskite is the first widely used 2D perovskite material. Kanatzidis et al. [58] first incorporated butylammonium (BA) into the perovskite to promote its vertical crystal growth on the substrate. They obtained a stable device with a PCE of 4.02% by adjusting the number of 2D inorganic layers. Chen et al. [59] studied the effect of the treatment with solvents of different polarities and boiling points on the crystallinity, crystal orientation, grain size and film quality of 2DRP perovskites. Dimethylacetamide (DMAC), which has a low polarity and a suitable boiling point, effectively accelerated the crystallization rate of the corresponding 2DRP perovskite. This yielded smooth, dense films with an efficiency of 12.15% and long charge-carrier lifetime, high photoluminescence intensity, low trap density and improved stability. However, the PCE of 2D perovskites is lower than that of their 3D counterparts because of the lower carrier mobility, the wide optical bandgap, the low conductivity and the large exciton binding energy. Therefore, Priya et al. [8] created a methylammonium (MA)-based 2D perovskite film by using the vapor-fumigation technique. The resulting films benefit from a higher conductivity, better carrier transport properties and a smaller exciton binding energy compared to traditional 2DRP perovskites based on bulky BA. Ultrastable 2DRP PSCs with a certified PCE of 16.6% were obtained. Their initially determined properties were degraded by only 2.2% after 1,512 hours without encapsulation when exposed to ambient conditions with 55% relative humidity (RH).

Very recently, Stoumpos et al. [60] reported a novel 2D perovskite that adopted the Dion–Jacobson (DJ) structure, which is considered to be more stable and more efficient than its RP counterpart. Here, the van der Waals gap between the organic layer and the inorganic slab is removed. The organic layers are connected with perovskite layers by strong hydrogen bonds in the 2D Dion–Jacobson (2DDJ) perovskite, which has a reasonably enhanced stability compared to the 2DRP perovskite. Guo et al. [19] developed a highly efficient and stable 1,3-propanediamine-based DJ phase perovskite with a maximum PCE of 13.3% by using the hot-casting method. Furthermore, the devices without encapsulation can retain more than 95% of their initial PCE upon exposure to ambient air for 4,000 hours, continuous light illumination for 3,000 hours and damp heat (heating at 85 °C and 85% RH) for 168 hours. These results demonstrate that PSCs based on DJ phase perovskites have higher efficiency and stability than those based on the RP structure, without the need for additives or pretreatment.

#### 2D/3D PSCs

Although many strategies have been proposed to improve the efficiency of 2D PSCs, their PCE is still not competitive with of the 3D devices. However, mixtures of 2D and 3D perovskites (2D/3D mixtures) inherit the remarkable stability of 2D perovskites while maintaining the excellent optoelectronic properties of 3D perovskites. 2D/3D perovskite materials can be obtained, for example, by incorporating alkylammonium salts of different size and hydrophobicity into 3D perovskites. However, this approach usually leads to perovskite materials of reduced moisture resistance.

Recently, Pan et al. [61] fabricated two new 2D/3D perovskite materials by incorporating hydrophobic alkylammonium salts with halogen functional groups (2-chloroethylammonium chloride and 2-bromoethylammonium bromide) into formamidinium-based 3D perovskites. By adjusting the relative amount of the ammonium salts the mixing ratio of the 2D and 3D perovskite phases could be controlled. The hydrophobic alkylammonium cations remarkably enhanced the humidity resistance of the 2D/3D mixtures. In addition, the halogen functional groups in the alkylammonium salts could cause changes of the electron cloud distribution and also enhance the hydrophobicity, which could improve the performance and the stability of the devices. Indeed, the resulting 2D/3D PSCs showed superior stability. Namely, the PCE of the devices decreased by only 8% of the initial value upon exposure to ambient conditions with 50 ± 5% RH for 2400 h. The maximum PCE of the devices reached 20.08% under standard AM1.5G illumination, while the hysteresis of the devices was reduced and the reproducibility of the properties was enhanced. Finally, this report reinforces recent expectations for further improvements of the stability and the efficiency of 2D/3D PSCs.

**Figure 4:**
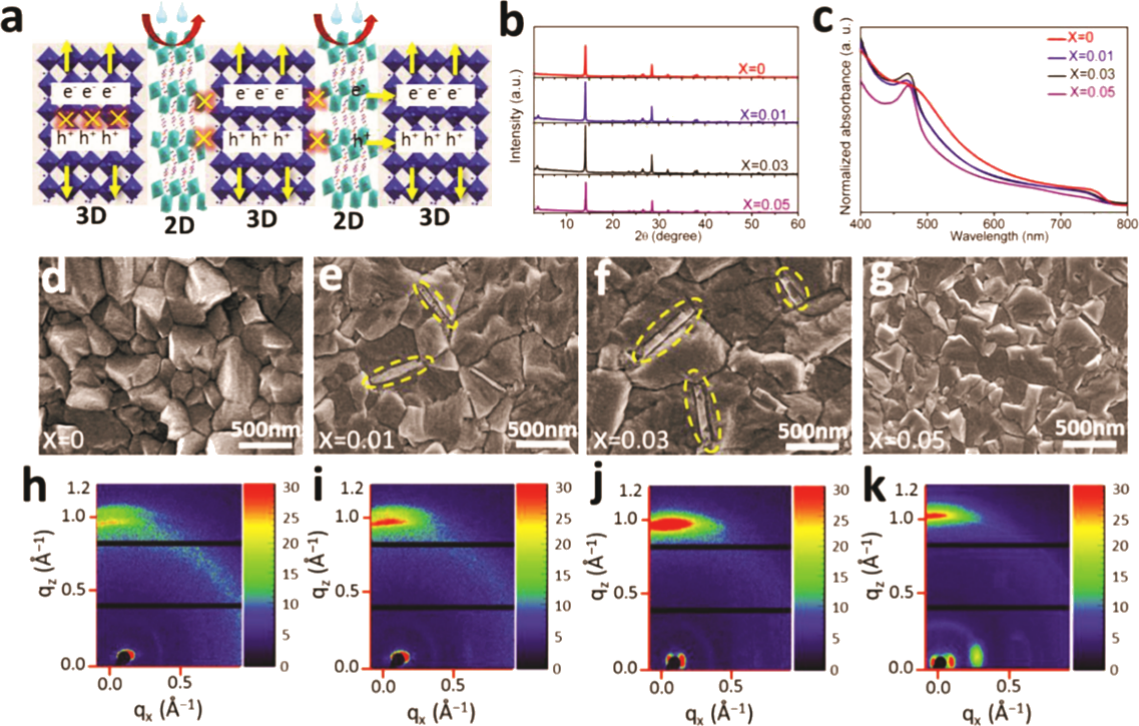
Structural characterization and morphology. (a) Schematic illustration of the structure of the 2D/3D PSCs. (b) XRD patterns as a function of the 2D perovskite doping concentration and (c) UV–vis absorption spectra of the (EDBEPbI_4_)*_x_*(MAPbI_3_)_1−_*_x_* films. (d–g) SEM images of perovskite films with different 2D concentrations. The “flake-like” structure in the yellow area is EDBEPbI_4_. (h–k) Grazing-incidence wide-angle X-ray scattering (GIWAXS) images of the films with different 2D concentrations. Reflections at *q*_z_ ≈ 1 is assigned to 3D perovskite (110). Reproduced with permission from [62], copyright 2018 John Wiley and Sons.

However, the 2D/3D perovskites prepared by the method mentioned above still suffer from lower partial charge-carrier extraction efficiency compared with the 3D devices. Xing et al. [62] introduced a new 2D/3D device with phase-segregated vertical heterojunctions of 2D and 3D perovskites (Figure 4a) by incorporating long C_6_H_18_N_2_O_2_PbI_4_ (EDBEPbI_4_) chains into 3D perovskites. The 2D/3D perovskites solved the discrepancy between PCE and stability. XRD patterns and UV–vis absorption spectra, retracing the formation of such heterojunctions, are shown in Figure 4b and Figure 4c. Doping with EDBEPbI_4_ gradually increases the grain size as shown in Figure 4d–g. The 2D perovskite vertically passivates the grain boundaries of the resulting 3D perovskite film. The photo-generated charge-carrier localization is minimized in the low-dimensional perovskite. Hence, the resulting phase pure 2D perovskite sections lead to an enhancement of the mobility, the lifetime and the vertical diffusion length of the charge carriers in the 2/3D perovskite. Figure 4h–k indicates that the 2D/3D perovskite (*x* = 0.03) has a better crystallinity than the pure 3D material because of its clearer crystal lattice phonon features. These factors greatly enhance the PCE of the corresponding 2D/3D PSCs, achieving a maximum PCE of 21.06% and a stabilized PCE of 19.66%. Moreover, these high-efficiency devices were of excellent stability, degrading by about 10% only after more than 3000 h in ambient air. Finally, the PCE of such optimized 2D/3D PSCs is already comparable to that of 3D PSCs. The successful preparation of highly efficient and stable 2D/3D PSCs provides the most reliable guarantee for the commercial application of PSCs, indicating that the era of PSCs is coming.

### Conclusion

This review summarizes the latest research on PCSs focusing on the perovskite layer. Over the past few years, many studies have reported highly efficient and low-cost PSCs, highlighting their extraordinary potential in photovoltaics. So far, efficiency enhancement, improvement of the stability and progress in large-area fabrication have laid the foundation for the commercialization of PSCs.

On the whole, the large-scale commercialization of PSCs is expected to help in solving the fossil fuel crisis and preventing the greenhouse effect. In particular, it can be incorporated into energy-saving architechture and intelligent environmentally friendly buildings. Obviously, the perovskite layer is of great significance to the efficiency, scalability and stability of PSCs. The development of hole and electron transport layers also plays an important role in the large-scale applicability of high-performance PSCs, especially in recent years when new organic interfacial materials continue to emerge [63]. Although some progress on the stability and scalability has been made, these two issues will still be the main challenges of commercializing PSCs in the future. In addition, the environmental impact of lead pollution from Pb-based perovskite materials also limits the large-scale commercialization of PSCs. To solve these critical issues, collaboration between different disciplines is needed. Finally, with all our efforts, the era of modern PSCs will surely come.

## References

[1] Zhang J, Hultqvist A, Zhang T, Jiang L, Ruan C, Yang L, Cheng Y, Edoff M, Johansson E M J (2017). ChemSusChem.

[2] Pham H D, Hayasake K, Kim J, Do T T, Matsui H, Manzhos S, Feron K, Tokito S, Watson T, Tsoi W C (2018). J Mater Chem C.

[3] Javaid S, Myung C W, Pourasad S, Rakshit B, Kim K S, Lee G (2018). J Mater Chem A.

[4] Wang Z, Kamarudin M A, Huey N C, Yang F, Pandey M, Kapil G, Ma T, Hayase S (2018). ChemSusChem.

[5] Han J, Yin X, Nan H, Zhou Y, Yao Z, Li J, Oron D, Lin H (2017). Small.

[6] Kosta I, Grande H, Tena-Zaera R (2017). Electrochim Acta.

[7] (2019). Best Research-Cell Efficiencies.

[8] Zhu X, Xu Z, Zuo S, Feng J, Wang Z, Zhang X, Zhao K, Zhang J, Liu H, Priya S (2018). Energy Environ Sci.

[9] Di Giacomo F, Shanmugam S, Fledderus H, Bruijnaers B J, Verhees W J H, Dorenkamper M S, Veenstra S C, Qiu W, Gehlhaar R, Merckx T (2018). Sol Energy Mater Sol Cells.

[10] Zong Y, Zhou Z, Chen M, Padture N P, Zhou Y (2018). Adv Energy Mater.

[11] Kim M, Kim G-H, Oh K S, Jo Y, Yoon H, Kim K-H, Lee H, Kim J Y, Kim D S (2017). ACS Nano.

[12] Chang C-W, Kwang Z-W, Hsieh T-Y, Wei T-C, Lu S-Y (2018). Electrochim Acta.

[13] Wang L-Y, Deng L-L, Wang X, Wang T, Liu H-R, Dai S-M, Xing Z, Xie S-Y, Huang R-B, Zheng L-S (2017). Nanoscale.

[14] Jeon N J, Noh J H, Kim Y C, Yang W S, Ryu S, Seok S I (2014). Nat Mater.

[15] Fujihara T, Terakawa S, Matsushima T, Qin C, Yahiro M, Adachi C (2017). J Mater Chem C.

[16] Fu F, Pisoni S, Weiss T P, Feurer T, Wäckerlin A, Fuchs P, Nishiwaki S, Zortea L, Tiwari A N, Buecheler S (2018). Adv Sci.

[17] Li C, Yin J, Chen R, Lv X, Feng X, Wu Y, Cao J (2019). J Am Chem Soc.

[18] Huang Y-C, Li C-F, Huang Z-H, Liu P-H, Tsao C-S (2019). Sol Energy.

[19] Ahmad S, Fu P, Yu S, Yang Q, Liu X, Wang X, Wang X, Guo X, Li C (2019). Joule.

[20] Ran C, Xi J, Gao W, Yuan F, Lei T, Jiao B, Hou X, Wu Z (2018). ACS Energy Lett.

[21] Gao L-L, Liang L-S, Song X-X, Ding B, Yang G-J, Fan B, Li C-X, Li C-J (2016). J Mater Chem A.

[22] Fan L, Ding Y, Luo J, Shi B, Yao X, Wei C, Zhang D, Wang G, Sheng Y, Chen Y (2017). J Mater Chem A.

[23] Xiao Z, Bi C, Shao Y, Dong Q, Wang Q, Yuan Y, Wang C, Gao Y, Huang J (2014). Energy Environ Sci.

[24] Mathies F, Abzieher T, Hochstuhl A, Glaser K, Colsmann A, Paetzold U W, Hernandez-Sosa G, Lemmer U, Quintilla A (2016). J Mater Chem A.

[25] Grancini G, Roldán-Carmona C, Zimmermann I, Mosconi E, Lee X, Martineau D, Narbey S, Oswald F, De Angelis F, Graetzel M (2017). Nat Commun.

[26] Conings B, Baeten L, De Dobbelaere C, D'Haen J, Manca J, Boyen H-G (2014). Adv Mater (Weinheim, Ger).

[27] Eperon G E, Burlakov V M, Docampo P, Goriely A, Snaith H J (2014). Adv Funct Mater.

[28] Gao C, Dong H, Bao X, Zhang Y, Saparbaev A, Yu L, Wen S, Yang R, Dong L (2018). J Mater Chem C.

[29] Li S, Zhang P, Chen H, Wang Y, Liu D, Wu J, Sarvari H, Chen Z D (2017). J Power Sources.

[30] Wang B, Zhang Z-G, Ye S, Rao H, Bian Z, Huang C, Li Y (2016). J Mater Chem A.

[31] Huang J, Wang M, Ding L, Yang Z, Zhang K (2016). RSC Adv.

[32] Paek S, Schouwink P, Athanasopoulou E N, Cho K T, Grancini G, Lee Y, Zhang Y, Stellacci F, Nazeeruddin M K, Gao P (2017). Chem Mater.

[33] Rai M, Rahmany S, Lim S S, Magdassi S, Wong L H, Etgar L (2018). J Mater Chem A.

[34] Li S-G, Jiang K-J, Su M-J, Cui X-P, Huang J-H, Zhang Q-Q, Zhou X-Q, Yang L-M, Song Y-L (2015). J Mater Chem A.

[35] Tang S, Deng Y, Zheng X, Bai Y, Fang Y, Dong Q, Wei H, Huang J (2017). Adv Energy Mater.

[36] Deng Y, Zheng X, Bai Y, Wang Q, Zhao J, Huang J (2018). Nat Energy.

[37] Zuo C, Vak D, Angmo D, Ding L, Gao M (2018). Nano Energy.

[38] Whitaker J B, Kim D H, Larson B W, Zhang F, Berry J J, van Hest M F A M, Zhu K (2018). Sustainable Energy Fuels.

[39] Chai G, Luo S, Zhou H, Daoud W A (2017). Mater Des.

[40] Huang H, Shi J, Zhu L, Li D, Luo Y, Meng Q (2016). Nano Energy.

[41] Jung Y-S, Hwang K, Heo Y-J, Kim J-E, Lee D, Lee C-H, Joh H-I, Yeo J-S, Kim D-Y (2017). ACS Appl Mater Interfaces.

[42] Hwang K, Jung Y-S, Heo Y-J, Scholes F H, Watkins S E, Subbiah J, Jones D J, Kim D-Y, Vak D (2015). Adv Mater (Weinheim, Ger).

[43] Barrows A T, Pearson A J, Kwak C K, Dunbar A D F, Buckley A R, Lidzey D G (2014). Energy Environ Sci.

[44] Das S, Yang B, Gu G, Joshi P C, Ivanov I N, Rouleau C M, Aytug T, Geohegan D B, Xiao K (2015). ACS Photonics.

[45] Kim J, Yun J S, Cho Y, Lee D S, Wilkinson B, Soufiani A M, Deng X, Zheng J, Shi A, Lim S (2017). ACS Energy Lett.

[46] Deng Y, Peng E, Shao Y, Xiao Z, Dong Q, Huang J (2015). Energy Environ Sci.

[47] Deng Y, Wang Q, Yuan Y, Huang J (2015). Mater Horiz.

[48] Wu W-Q, Yang Z, Rudd P N, Shao Y, Dai X, Wei H, Zhao J, Fang Y, Wang Q, Liu Y (2019). Sci Adv.

[49] Ciro J, Mejía-Escobar M A, Jaramillo F (2017). Sol Energy.

[50] Kim J-E, Jung Y-S, Heo Y-J, Hwang K, Qin T, Kim D-Y, Vak D (2018). Sol Energy Mater Sol Cells.

[51] Jeong D-N, Lee D-K, Seo S, Lim S Y, Zhang Y, Shin H, Cheong H, Park N-G (2019). ACS Energy Lett.

[52] Bella F, Griffini G, Correa-Baena J-P, Saracco G, Gratzel M, Hagfeldt A, Turri S, Gerbaldi C (2016). Science.

[53] Zhang F, Wang S, Zhu H, Liu X, Liu H, Li X, Xiao Y, Zakeeruddin S M, Grätzel M (2018). ACS Energy Lett.

[54] Jeon N J, Na H, Jung E H, Yang T-Y, Lee Y G, Kim G, Shin H-W, Il Seok S, Lee J, Seo J (2018). Nat Energy.

[55] Quan L N, Yuan M, Comin R, Voznyy O, Beauregard E M, Hoogland S, Buin A, Kirmani A R, Zhao K, Amassian A (2016). J Am Chem Soc.

[56] Koh T M, Shanmugam V, Schlipf J, Oesinghaus L, Müller-Buschbaum P, Ramakrishnan N, Swamy V, Mathews N, Boix P P, Mhaisalkar S G (2016). Adv Mater (Weinheim, Ger).

[57] Stoumpos C C, Cao D H, Clark D J, Young J, Rondinelli J M, Jang J I, Hupp J T, Kanatzidis M G (2016). Chem Mater.

[58] Cao D H, Stoumpos C C, Farha O K, Hupp J T, Kanatzidis M G (2015). J Am Chem Soc.

[59] Qiu J, Zheng Y, Xia Y, Chao L, Chen Y, Huang W (2019). Adv Funct Mater.

[60] Mao L, Ke W, Pedesseau L, Wu Y, Katan C, Even J, Wasielewski M R, Stoumpos C C, Kanatzidis M G (2018). J Am Chem Soc.

[61] Liu G, Zheng H, Xu X, Xu S, Zhang X, Pan X, Dai S (2019). Adv Funct Mater.

[62] Li P, Zhang Y, Liang C, Xing G, Liu X, Li F, Liu X, Hu X, Shao G, Song Y (2018). Adv Mater (Weinheim, Ger).

[63] Pham H D, Li X, Li W, Manzhos S, Kyaw A K K, Sonar P (2019). Energy Environ Sci.

